# Integrated bioinformatics and validation reveal TMEM45A in systemic lupus erythematosus regulating atrial fibrosis in atrial fibrillation

**DOI:** 10.1186/s10020-025-01162-0

**Published:** 2025-03-18

**Authors:** Hongjie Xu, Sufan Ding, Xiaoping Ning, Ye Ma, Qi Yu, Yi Shen, Lin Han, Zhiyun Xu

**Affiliations:** 1 Department of Cardiovascular Surgery, Changhai Hospital, The Naval Medical University, 168 Changhai Road, Shanghai, 200433 China; 2 National Clinical Research Center of Kidney Diseases, Jinling Hospital, Nanjing University School of Medicine, Nanjing, 210002 Jiangsu China; 3 Department of Cardiothoracic Surgery, Jinling Hospital, Medical School of Nanjing University, Nanjing, 210002 Jiangsu China

**Keywords:** Systemic lupus erythematosus, Atrial fibrillation, Bioinformatics, TMEM45A, Fibrosis

## Abstract

**Background:**

Accumulative evidence has shown that systemic lupus erythematosus (SLE) increases the risk of various cardiovascular diseases including atrial fibrillation (AF). The study aimed to screen potential key genes underlying co-pathogenesis between SLE and AF, and to discover therapeutic targets for AF.

**Methods:**

Differentially expressed genes (DEGs) were identified, and co-expressed gene modules were obtained through weighted gene co-expression network analysis (WGCNA) based on the AF and SLE expression profiles from the GEO database. Subsequently, machine learning algorithms including LASSO regression and support vector machine (SVM) method were employed to identify the candidate therapeutic target for SLE-related AF. Furthermore, the therapeutic role of TMEM45A was validated both in vivo and vitro.

**Results:**

Totally, 26 DEGs were identified in SLE and AF. The PPI network combined with WGCNA identified 51 key genes in SLE and AF. Ultimately, Machine learning-based methods screened three hub genes in SLE combined with AF, including TMEM45A, ITGB2 and NFKBIA. The cMAP analysis exposed KI-8751 and YM-155 as potential drugs for AF treatment. Regarding TMEM45A, the aberrant expression was validated in blood of SLE patients. Additionally, TMEM45A expression was up-regulated in the atrial tissue of patients with AF. Furthermore, TMEM45A knockdown alleviated AF occurrence and atrial fibrosis in vivo and Ang II-induced NRCFs fibrosis in vitro.

**Conclusion:**

The crosstalk genes underlying co-pathogenesis between SLE and AF were unraveled. Furthermore, the pro-fibrotic role of TMEM45A was validated in vivo and vitro, highlighting its potential as a therapeutic target for AF.

**Supplementary Information:**

The online version contains supplementary material available at 10.1186/s10020-025-01162-0.

## Introduction

Atrial fibrillation (AF) is the most common cardiac arrhythmia, characterized by disordered atrial contraction, leading to electronic and structural atrial remolding (Brundel et al. 2022). The incidence of AF can cause severe complications including stroke, heart failure, and even cardiac arrest, resulting in increased morbidity and mortality (Zoni-Berisso et al. 2014). Although AF primarily occurs in older individuals and those with unhealthy lifestyles, it can also be triggered by inflammatory situations including systemic lupus erythematosus (SLE) (Hindricks et al. 2021). SLE is a typical autoimmune rheumatic disease characterized by multisystem involvement, particularly affecting the cardiovascular system (Chen et al. 2022). Previous studies have indicated that SLE may increase the risk of cardiovascular diseases, such as coronary artery disease, conduction system disorder and heart failure. A meta-analysis revealed that the incidence of AF in SLE patients reaches 1.15%, and confirmed that the prevalence of AF events is significantly higher in SLE cohorts (Chen et al. 2022). However, compared with other cardiovascular diseases, there is limited evidence focusing on the mechanism of crosstalk between SLE and AF. Therefore, identifying the potential underlying mechanism in SLE patients with AF is indispensable.

As an archetypal autoimmune disease, SLE can initiate systemic inflammation and immune response. Previous study has proposed that certain antibodies may cause myocarditis and arrythmias via targeting cardiomyocytes (Monti et al. 2016). Meanwhile, the release of various serum proinflammatory factors (IL-2, IL-6, TNF-α and C-reactive protein) was in the development of cardiovascular diseases in SLE (Zhang et al. 2019). Though the pathophysiological mechanisms of AF have not been fully elucidated, systemic inflammation may fuel the atrial electrical and structural remolding, which determine the pathophysiology basis of AF (Chen et al. 2022). Thus, unveiling the specific molecular mechanism underlying pathophysiology of AF in SLE could provide insights into the diagnosis, prevention and treatment of AF in SLE. Nevertheless, there are few related scientific studies focused on the investigation and identifying the occurrence and development of AF in SLE.

In the present study, multiple integrative bioinformatics tools were employed to unveil the potential hub genes involving biological processes in AF combined with SLE. The diagnostic markers of AF in SLE were identified, and their relationship with immune infiltration was explored. Furthermore, the role of the screened hub gene was validated. Thus, the study aimed to investigate the diagnostic markers and potential therapeutic targets of AF in SLE.

## Materials and methods

### Microarray data collecting and processing

Four raw datasets of AF and control groups, including GSE14975, GSE31821, GSE41177 and GSE79768 were obtained from GEO database (https://www.ncbi.nlm.nih.gov/geo/). The GSE14975 datasets contained transcriptome analyses of left atrium from 5 AF patients and 5 individuals with sinus rhythm. The GSE31821 datasets identified 4 left atrium samples from patients with AF and 2 left atrium samples from people with sinus rhythm. The GSE41177 datasets contained 19 left atrium samples, including 16 left atrium tissues from AF patients and 3 left atrium tissues from people with sinus rhythm. In GSE79768 datasets, left atrium samples from 6 individuals with sinus rhythm and 7 AF patients were identified. Subsequently, four datasets of AF were integrated after batch correction based on the combat function of the “SVA” package. The datasets related to SLE (GSE50772) were obtained from GEO datasets. In GSE50772, the transcriptome analyses from peripheral blood mononuclear cells (PBMCs) from 20 healthy individuals and 61 SLE patients. The simplified workflow was depicted in Supplementary Fig. 1.

### Differentially expressed genes (DEGs) analysis

After normalization, the DEGs of AF and SLE were identified from integrated AF datasets and GSE50772 through the “limma” package. And the threshold was defined as |log2FC|> 1.0 and p < 0.05. In addition, the expression files of DEGs were visualized through the “ggplot2” and the “pheatmap” packages respectively. Furthermore, the overlapping of DEGs from AF and SLE was performed to identity through an online Venn diagram generator (https://bioinfogp.cnb.csic.es/tools/venny).

### Weighted gene co-expression network analysis (WGCNA) and key module genes identification

Based on the DEGs from AF and SLE datasets, the co-expressed key modules were screened through WGCNA. Firstly, the flashCluster program was employed to perform the hierarchical clustering analysis of samples to discover and eliminate outliers. Then, the appropriated soft threshold power was generated and used to design a physiologically important scale-free network. Thirdly, a dynamic tree-cutting technique was used to create a topological overlap matrix (TOM) based on the adjacency matrix to detect gene modules. Finally, gene significance (GS) and module membership (MM) were determined to evaluate the correlation between modules and clinical traits. Then, the genes in most related gene modules emerged for further PPI network analysis.

### The construction of protein–protein interaction (PPI) network

To evaluate the interaction between key module genes in AF with SLE, the STRING database was used to construct the PPI network (https://string-db.org/). The combined score threshold was defined as 0.9. Furthermore, the PPI network was visualized through Cytoscape software. Moreover, four network properties (degree, stress, betweenness and closeness) were calculated to reflect the importance of each protein. The key module genes were generated from the overlapping of the top 50 proteins in four network properties.

### Functional enrichment analysis

The key module genes from PPI network and DEGs in AF with SLE were merged to explore the biological function and concrete mechanism through Gene Ontology (GO) and Kyoto Encyclopedia of Genes and Genomes (KEGG) pathway enrichment analysis. The “clusterprofiler” package was used to perform enrichment analysis, and the higher Gene Ratio was considered more significant.

### Connectivity map (cMAP) analysis

The up-regulated core genes merged from DEGs of AF with SLE and key modules genes were incorporated cMAP online (https://clue.io) datasets (Subramanian et al. 2017), and the potential small molecular drugs for the treatment of AF with SLE. Furthermore, the top 10 compounds were identified and presented.

### Machine learning

To further identify the hub genes in AF with SLE, two types of machine learning analysis were performed. The least absolute shrinkage and selection operator (LASSO) algorithm and support vector machine (SVM) method were performed to select hub genes in AF and SLE respectively. Briefly, the hub genes of AF and SLE were generated with the intersection of LASSO and SVM analysis respectively, and the overlapping was deemed as hub genes in AF with SLE.

### Immune infiltration analysis

The immune cell filtration degree in AF and SLE were analyzed through single sample Gene Set Enrichment Analysis (ssGSEA) respectively. By the “GSVA” package, the differences in immune cell content filtration in different groups were explored. Furthermore, the correlation between the immune cell content and the hub gene expression was determined.

### GSEA analysis of transmembrane protein 45A (TMEM45A)

The Gene Set Enrichment Analysis (GSEA) tool was employed to explore the molecular signaling pathway. The c2.cp.kegg.v7.4.entrez.gmt gene sets obtained from the official website were used for pathway enrichment analysis.

### Histological staining

The left atrium samples were collected from patients with or without persistent AF receiving valvular surgery. As shown in supplementary Table 1, there were no significant differences in clinical features between the two groups. The samples were fixed with paraformaldehyde, and then embedded with paraffin. Subsequently, 5 µm sections were prepared for hematoxylin & eosin (HE) staining for pathological evaluation, or Masson staining with Masson staining kit (Solarbio, China). The immunohistochemistry (IHC) analysis was stained according to the previous procedure and visualized by 3, 3-diaminobenzidine. The human samples were obtained with written informed consent from patients or their families. All experiment was approved by the Ethics Committee of Changhai hospital on human research.

### The Elisa experiment of TMEM45A

The blood serum from normal and SLE patients was collected, and the level of TMEM45A was detected through Elisa regent kit of TMEM45A (Weiaobio, shanghai) according to the protocol.

### Animal experiments

Sprague–Dawley (SD) male rats (weighted 250 mg) were purchased from Model Organisms (Shanghai, China). The animals were kept in a dedicated specific pathogen-free animal center at the Changhai Hospital. Changhai Hospital's Animal Care and Use Committee granted its approval for every experiment.

The AF model was established according to previous study (Lv et al. 2019). The rats were randomly divided into 4 groups (9 rats in each group). (1) Negative control (NC) group, wherein rats were injected with 0.9% saline through the tail vein for 7 days; (2) AF group, wherein rats were injected with acetylcholine (ACh)-CaCl2 (60 μg/ml ACh and 10 mg/ml CaCl2) daily via the tail vein at 1 ml/kg for 7 days; (3) AF + AAV-shRNA-NC group, wherein rats were injected with 50ul AAV-shRNA-NC (4*10^12^ vg/ml) through right jugular vein priorly. After 3 days, the rats were injected with acetylcholine (ACh)-CaCl2 daily via the tail vein at 1 ml/kg for 7 days. (4) AF + AAV-shRNA-TMEM45A, wherein rats were injected with 50ul AAV-shRNA-TMEM45A (4*10^12^ vg/ml) through right jugular vein priorly. Then the rats were injected with acetylcholine (ACh)-CaCl2 continuously via the tail vein at 1 ml/kg for 7 days after 3 days of infection. Subsequently, the rats were anesthetized through isoflurane, and the hearts were obtained after electrocardiogram was recorded. The pscAAV2/9-U6-shRNA and pscAAV2/9-U6-shRNA (TMEM45A) were constructed by Obio Technology (Shanghai). It is noted that scAAV is a kind of self-complementary AAV, which could enable the protein expression to reach the peak after 3 days of infection (McCarty 2008).

### Electrocardiogram recording and analysis

Electrocardiogram (a standard II lead was recorded) of rats and AF duration were recorded using MedLab-U/4C501H bio-signal collection system. The rats were deeply anesthetized with isoflurane and placed on the console. The AF was induced by injection of Ach-CaCl2 mixture before recording. The typical AF electrocardiogram, manifested by the disappearance of the P wave and emergence of F wave were the markers of AF occurrence, while the resumption of sinus rhythm, appearance of the P wave, and disappearance of F wave were markers of AF termination, and the time from AF occurrence to termination was AF duration.

### The isolation of neonatal rat cardiac fibroblasts (NRCFs)

The NRCFs were isolated from neonatal rats as previously described (Zhao et al. 2018). The hearts of neonatal rats were obtained and cut into about 1 mm^3^. Then the hearts were digested with 0.2% trypsin and 0.1% collagenase II and certificated. The cells were collected in DMEM culture medium with 10% fetal bovine serum. The NRCFs were obtained through differential adherence method for 40 min.

### The transfection of siRNA

To knockdown the expression of TMEM45A in NRCFs, the small interfering RNA (siRNA)-TMEM45A (si-TMEM45A) and negative control (si-NC) were designed and synthesized by IBSBIO (Shanghai, China). The sequence of si-TMEM45A was listed as follows: siRNA (376–394) 5′-GGACTTTCTTCATCATGAT-3′. The NRCFs were transfected with siRNA through jetPRIME regent (univ, shanghai) according to the manufacturer’s instructions.

### Western blotting (WB) experiments

Total proteins were extracted with SDS lysis (Beyotime, China) from NRCFs. Different protein samples were separated by SDS-PAGE and transferred into polyvinylidene difluoride membrane. Subsequently, the membrane was incubated with primary antibody and corresponding secondary antibody respectively. The immunoreactive bands were visualized by chemiluminescence (Thermo Scientific) and evaluated by Image J software. β-actin was used as the control.

### Cell proliferation assay

After treatment, NRCFs were incubated with 100 ul 10% CCK-8 solution (Beyotime) diluted by cell culture medium for 30 min, and OD values at 450 nm were detected by microplate reader.

The EdU incorporation assay in vitro was performed according to the instruction of Cell Proliferation kit (Beyotime). And the positive cells were observed through fluorescence microscope.

### Cell migration assay

The migration of NRCFs was assessed through Transwell cell invasive assay. Cells were seeded in the 8-μm pore size chambers with transparent polyester. After 24 h of incubation, cells were migrated to the bottom of the chambers and stained with crystal violet (0.1%).

### Statistical analysis

The statistical analysis was performed in GraphPad Prim 8 software. Statistical differences between the two groups were analyzed by a two-tailed Student's t-test (normal distribution) or the Mann–Whitney U test (non-normal distribution). Statistical differences between multiple groups were analyzed by one-way ANOVA followed by the post hoc Tukey test (homogeneity of variance) or one-way ANOVA followed by the Dunnett's T3 test (heterogeneity of variance). A p-value of < 0.05 was considered statistically significant.

## Results

### Identification of DEGs in AF with SLE

**Fig. 1:**
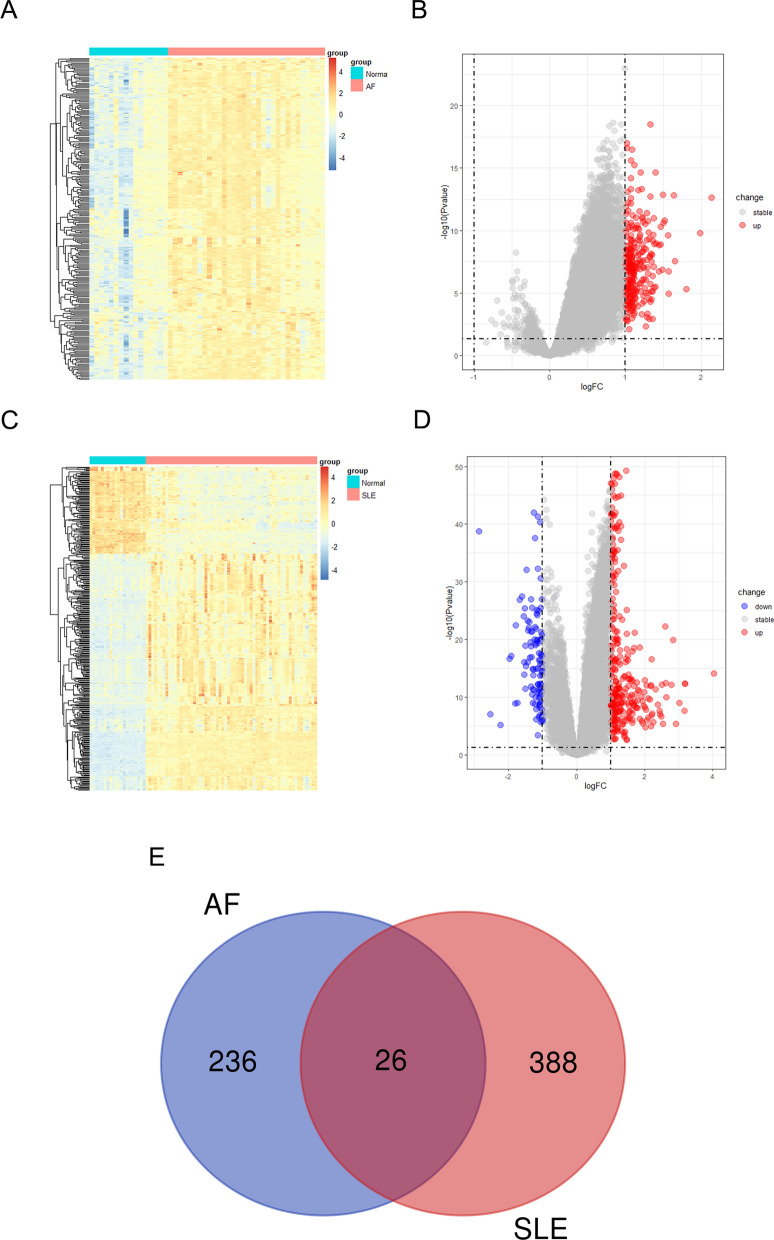
DEGs identification from AF and SLE. **A** The heatmap of AF DEGs analysis results based on merged datasets including GSE14975, GSE31821, GSE41177 and GSE79768. **B** The volcano plot of AF DEGs analysis results based on merged datasets including GSE14975, GSE31821, GSE41177 and GSE79768. **C** The heatmap of SLE DEGs analysis results based on GSE50772. **D** The volcano plot of SLE DEGs analysis results based on merged datasets including GSE50772. **E** Identification of 26 overlapping genes between DEGs of AF and SLE

Four raw datasets of left atrial samples from AF and control patients in GEO database were extracted and combined after carrying out batch effect removal. As shown in Supplementary 2A, B, the differences among the four datasets were significantly decreased after batch effect removal. Totally, 262 differential expressed genes (DEGs) were identified, with all genes being up-regulated. The volcano plot and the heatmap were depicted to reflect the expression pattern of DEGs in integrated AF datasets (Fig. 1A, B). Moreover, we obtained 414 SLE-related DEGs in GSE50772, of which 302 genes were up-regulated and 112 genes were down-regulated, as shown in the heatmap and volcano plot (Fig. 1C, D). Furthermore, 26 common genes were identified between AF and SLE-related DEGs after intersection (Fig. 1E**)**. The expression patterns of the 26 DEGs in AF and SLE datasets were exhibited in Supplementary Table 2.

### Identification of co-expression gene modules

**Fig. 2:**
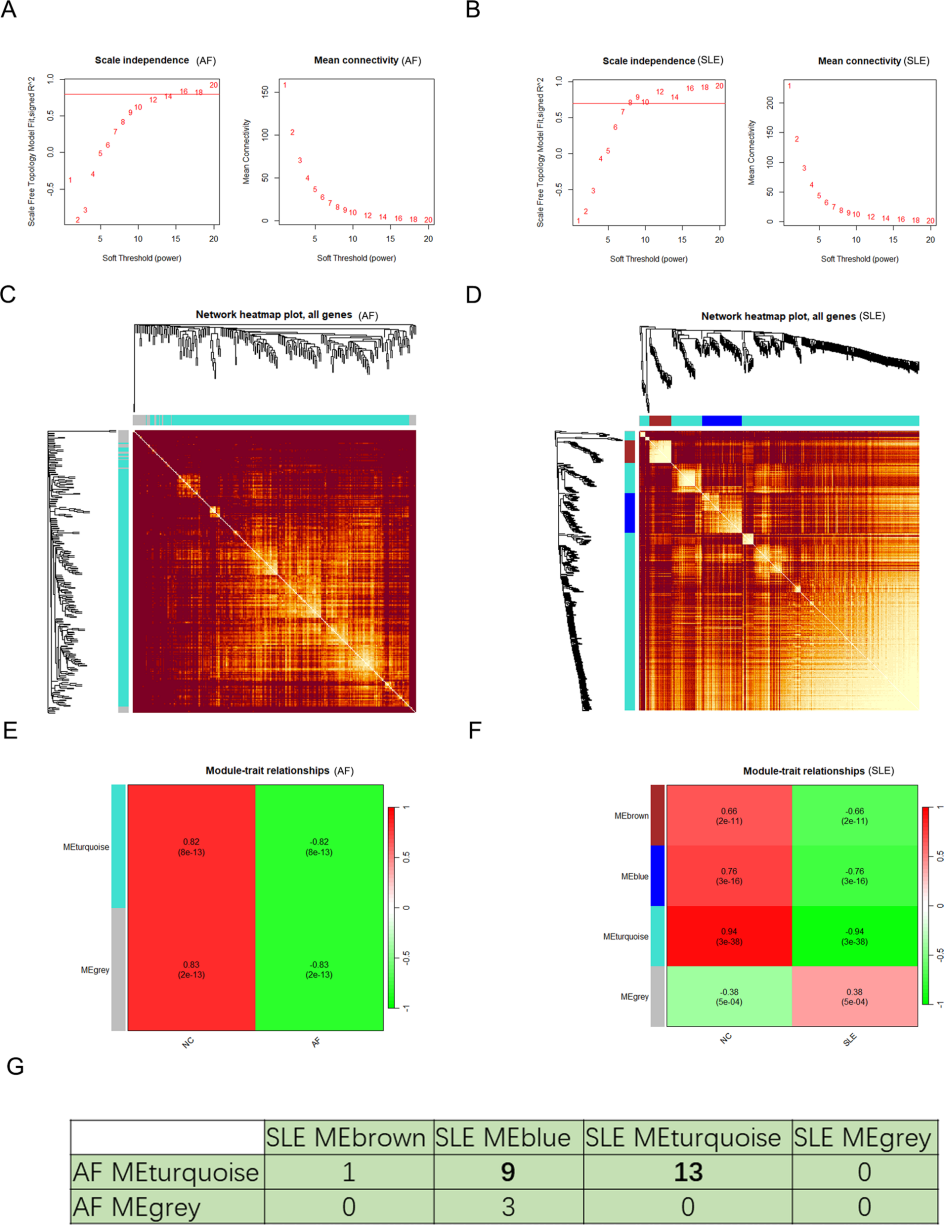
Weighted co-expressed network analysis and related key gene modules construction. **A**, **B** Analysis of scale-free topology model to identify the appropriate soft threshold based on the average connectivity and scale independence in AF and SLE. **C**, **D** The network heatmap of gene dendrogram and module eigengenes in AF and SLE. **E**, **F** The heatmap of correlation between module eigengenes and status of AF and SLE respectively. The correlation and *p* value were presented. **G** Number of intersecting genes of each key module of AF and SLE

To further explore the key genes in AF and SLE, WGCNA was performed to identify the relevant gene modules in DEGs of AF and SLE respectively. According to the scale independence and average connectivity, soft-thresholding power of 14 and 8 were selected for AF and SLE datasets respectively (Fig. 2A, B). DEGs in AF were clustered into 2 modules while DEGs in SLE were clustered into 4 modules through hierarchical clustering analysis and dynamic branch cut methods for the gene dendrograms (Fig. 2C, D). To identify the key modules related to AF and SLE, correlations between gene modules and clinical traits in AF and SLE were explored respectively (Fig. 2E, F). Furthermore, it was found that two SLE-related modules (SLE-MEblue and SLE-MEturquoise) and AF-MEturquoise module shared 9 and 13 genes respectively (Fig. 2G). Thus, these modules were identified as co-expressed gene modules closely related to AF with combined SLE. Finally, 287 genes were obtained after merging genes from SLE-MEblue and AF-MEturquoise, and 545 genes were obtained after merging genes from SLE-MEturquoise and AF-MEturquoise.

### The PPI network of key genes

**Fig. 3:**
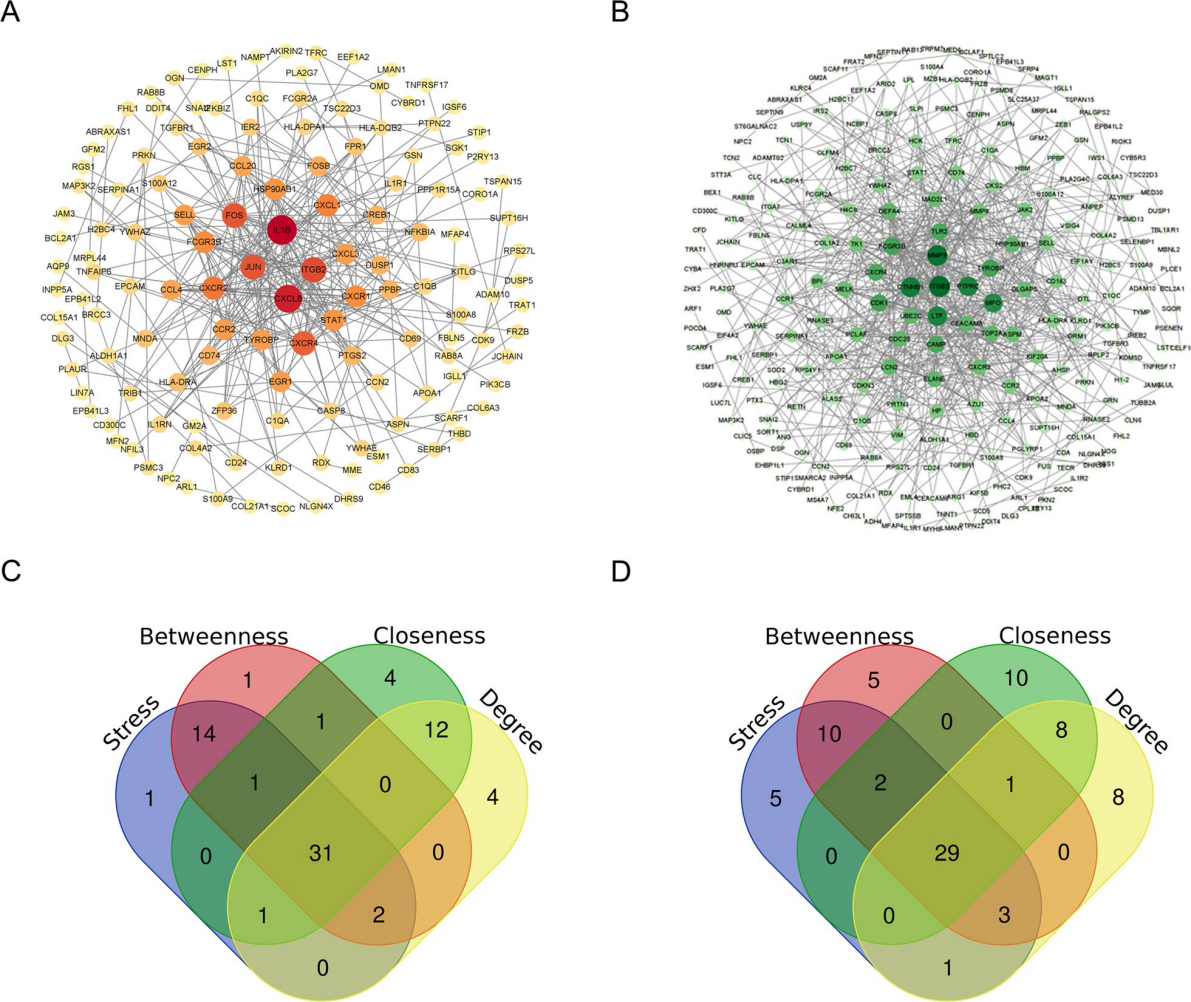
Key module genes identification based on the PPI network analysis in AF and SLE. **A** The PPI network composed of genes in SLE-MEblue module and AF-MEturquoise module. **B** The PPI network was composed of genes in SLE-MEturquoise module and AF-MEturquoise module. **C** Screening 31 key module genes in the PPI network of SLE-MEblue module and AF-MEturquoise module based on Stress, Betweenness, Closeness, and Degree. **D** Screening 29 key module genes in the PPI network of SLE-MEturqoise module and AF-MEturquoise module based on Stress, Betweenness, Closeness, and Degree

The interaction data of 287 genes including genes in co-expressed gene modules (SLE-MEblue and AF-MEturquoise) were obtained from STRING database, and the PPI network was visualized through Cytoscape (Fig. 3A). Similarly, the interaction of 545 genes involving all genes (SLE-MEturquoise and AF-MEturquoise) were obtained from STING database and then imported into Cytoscape to visualize the PPI network (Fig. 3B). Subsequently, the top 50 important nodes from each network property were screened. As shown in Fig. 3C, D, 31 top genes were obtained from co-expressed gene modules (SLE-MEblue and AF-MEturquoise), and 29 top genes were identified from co-expressed gene modules (SLE-MEturquoise and AF-MEturquoise). Finally, 51 hub genes were identified based on the PPI network after merging.

### GO and KEGG pathways enrichment analysis

**Fig. 4:**
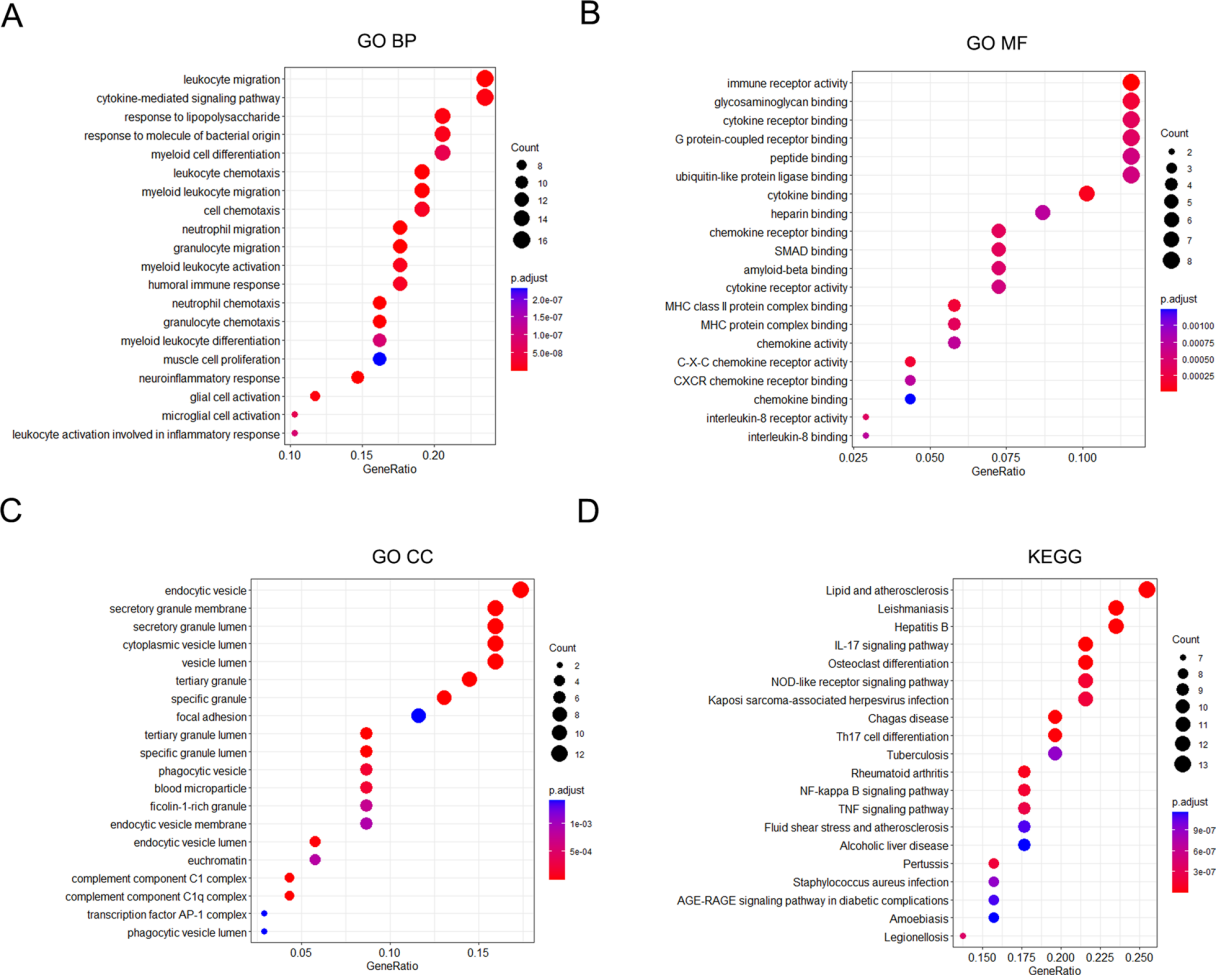
GO and KEGG pathway analysis for core genes merged from 26 DEGs and 51 key modules genes of PPI network. **A** The first 20 significantly enriched GO annotation of BP. **B** The 20 first significantly enriched GO annotation of MF. **C** The first 20 significantly enriched GO annotation of CC. **D** The first 20 significantly enriched KEGG pathways

Subsequently, 51 hub genes obtained from the PPI network were merged with 26 DEGs shared by SLE and AF, and 69 genes were identified as core genes of AF associated with SLE for further analysis finally. To elucidate the function and specific mechanisms of the core genes in AF associated with SLE, functional enrichment and KEGG analysis were performed. In the domain of BP, the core genes showed significant enrichment in leukocyte migration, cytokine-mediated signaling pathway and response to lipopolysaccharide **(**Fig. 4A). In terms of MF, immune receptor activity, glycosaminoglycan and cytokine receptor binding emerged as the most relevant items (Fig. 4B). Pertaining to CC, the core genes were predominantly localized in the endocytic vesicle, secretory granule membrane and secretory granule lumen (Fig. 4C). Furthermore, the result of KEGG analysis showed that core genes were closely associated with lipid and atherosclerosis, leishmaniasis and hepatitis B (Fig. 4D).

### Identification of potential small-molecular compounds for treatment

**Fig. 5:**
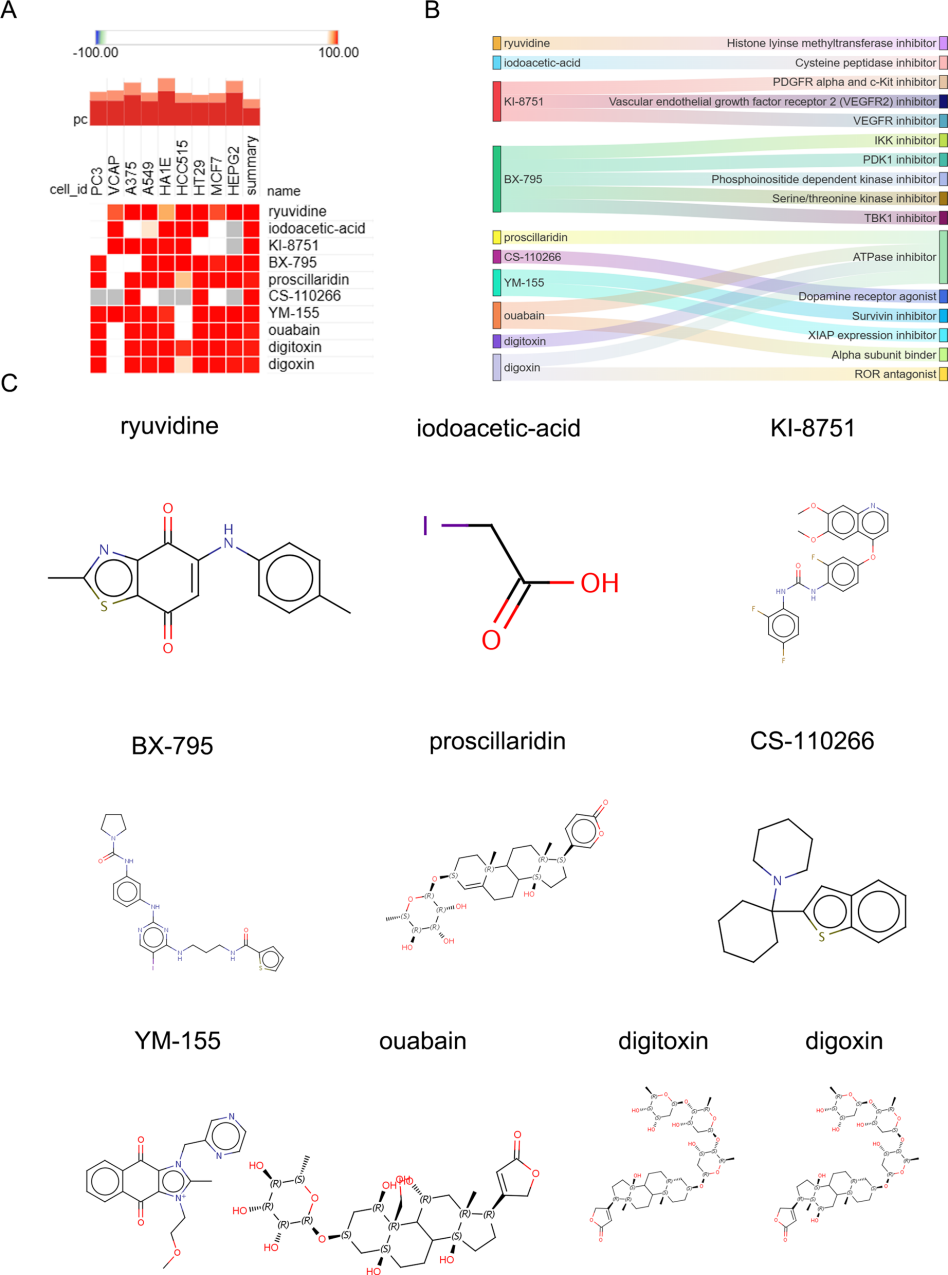
Screening potential small-molecular therapeutic compounds for AF with SLE through cMAP analysis. **A** The heatmap of the top 10 small-molecular compounds with the most significant negative enrichment scores in 10 cell lines. **B** The description of the targeted pathways of the top 10 small-molecular compounds. **C** The chemical structures of the top 10 small-molecular compounds

To further identify potential small-molecular compounds that might have therapeutic effects on AF with SLE, 69 core genes (up-regulated in AF) as identified previously, were imported into the connectivity map (cMAP) datasets to explore effective drugs. As shown in Fig. 5A, the top 10 small-molecular compounds including ryuvidine, iodoacetic-acid, KI-8751, BX-795, proscillaridin, CS-110266, YM-155, ouabain, digitoxin and digoxin, were predicted to have the highest scores and play a pharmacological role in the treatment of AF with SLE. Description of the targeted pathways and the chemical structures of the top 10 small-molecular compounds were presented in Fig. 5B, C.

### Identify the key genes in AF with combined SLE

**Fig. 6:**
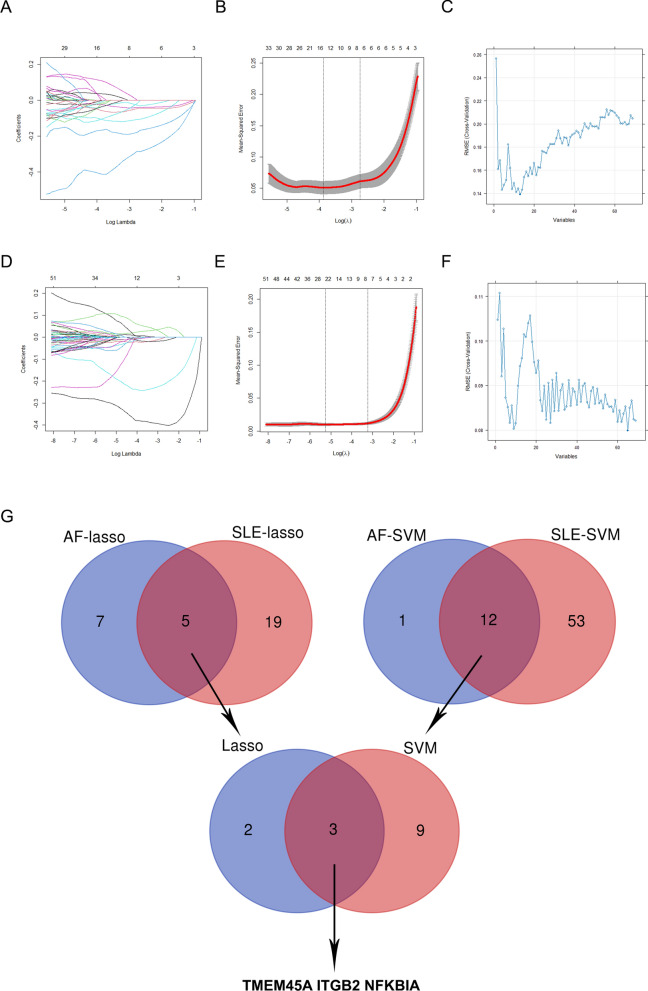
Screening hub genes based on the machine learning methods in AF and SLE. **A** Coefficient profiles of variables in LASSO regression model in AF. **B** Ten fold cross-validation for turning parameter. **C** The optimum root mean squared error (RMSE) of SVM-based method based on 13 characteristic genes in AF. **D** Coefficient profiles of variables in LASSO regression model in SLE. **E** Ten fold cross-validation for turning parameter. **F** The optimum root mean squared error (RMSE) of SVM-based method based on 65 characteristic genes in SLE. **G** Venn diagram showed that 5 common genes were selected by LASSO regression model and 12 common genes were screened by SYM-based method. And 3 hub genes in AF with SLE were finally identified after intersection

Based on the 69 core genes in AF combined with SLE, the LASSO regression model and SVM method were employed for identifying the final hub genes. As illustrated in Fig. 6A–C, LASSO regression identified 12 genes in the model from AF, and the SMV method screened 13 genes in AF. Similarly, 24 genes and 65 genes related to SLE were identified by LASSO regression and SVM method respectively (Fig. 6D–F). After intersection analysis, 3 common candidate genes (TMEM45A, ITGB2 and NFKBIA) were identified as hub genes for AF with SLE (Fig. 6G). The expression patterns of the 3 hub genes in AF datasets, SLE datasets and merged AF datasets were exhibited in Supplementary Tables 3, 4, 5 respectively.

### Immune cell correlation analysis

Based on the result of GO and KEGG analysis, it was found that the function and pathways of core genes in AF with SLE were closely related to inflammatory and immune activity. Thus, ssGSEA analysis was performed to characterize immune cell profiles in AF and SLE datasets. As shown in Supplementary Fig. 3A, B, the scores of immune cell contents were higher in most AF groups, with 17 immune cells (activated CD4 T cell, activated dendritic cell, central memory CD4 T cell, central memory CD8 T cell, effector memory CD4 T cell, effector memory CD8 T cell, immature B cell, immature dendritic cell, macrophage, mast cell, MDSC, memory B cell, monocyte, natural killer cell, neutrophile, regulatory T cell and type 1 T helper cell) were significantly increased in AF groups. However, the abundance of immune cell infiltration in SLE was different, with 10 immune cells (activated CD4 T cell, activated dendritic cell, central memory CD8 T cell, eosinophil, immature dendritic cell, macrophage, mast cell, MDSC, neutrophile and plasmacytoid dendritic cell) increased significantly in SLE, whereas 7 immune cells (activated CD8 T cell, CD56 dim natural killer cell, effector memory CD4 T cell, effector memory CD8 T cell, nature killer T cell, type 17 T helper cell and type 2 T helper cell) decreased significantly in SLE (Supplementary Fig. 4A, 3B). Furthermore, the correlation between the expression of 3 hub genes and immune cell infiltration in AF and SLE was explored through Pearson’s correlation coefficient. As presented in Supplementary Fig. 3C–E, the expression of ITGB2 and NFKBIA were positively correlated with central memory CD8 T cells in AF. The expression of TMEM45A was positively correlated with mast cells in AF. In SLE, the expression of ITGB2, NFKBIA and TMEM45A were all positively correlated with activated dendric cells (Supplementary Fig. 4C–E).

### The GSEA of TMEM45A in AF

**Fig. 7:**
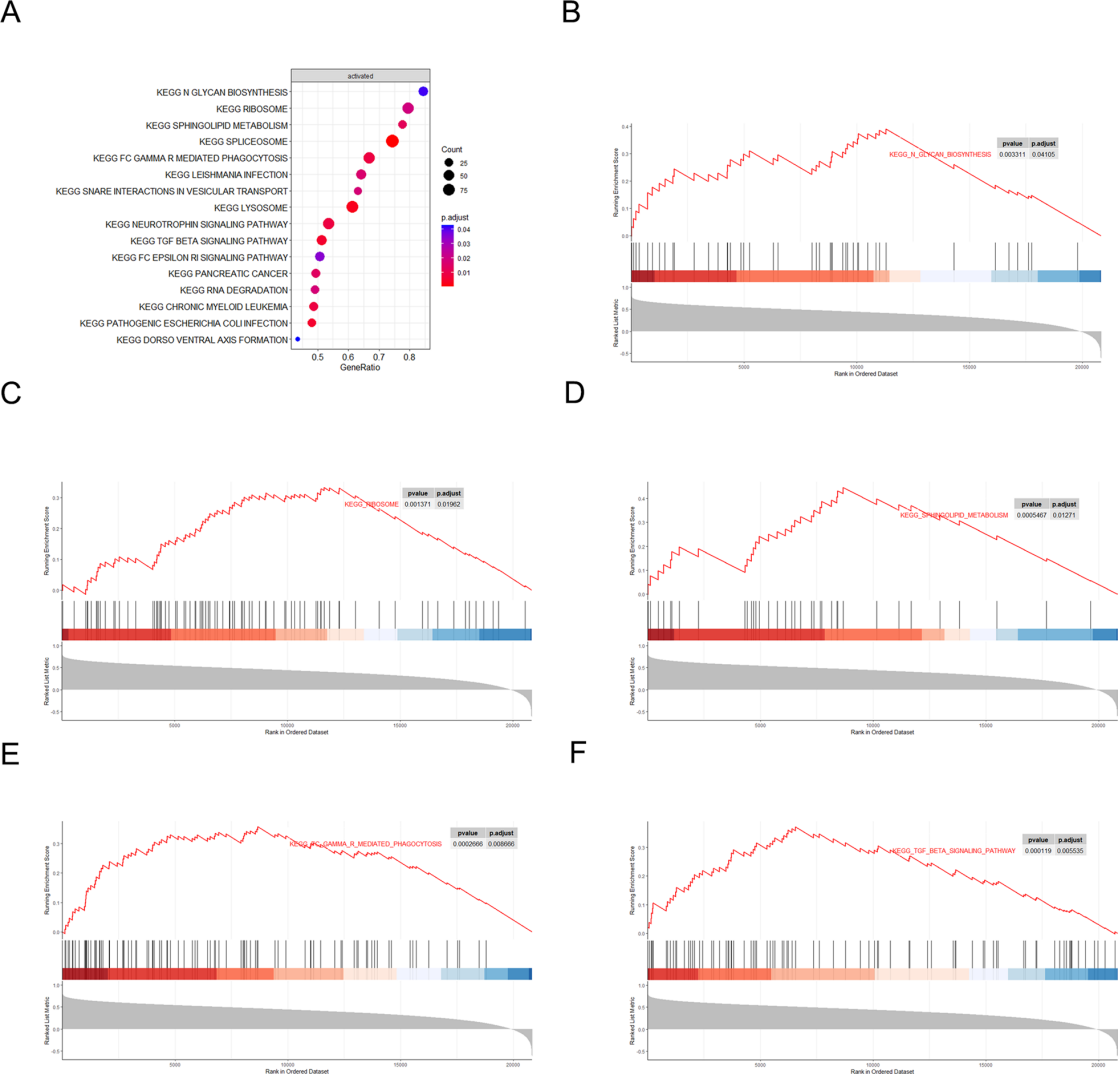
The GSEA analysis of TMEM45A in AF. **A** The GSEA analysis showed the KEGG pathways enrichment of TMEM45A in AF. **B**–**F** The specific enrichment pathway including “KEGG N GLYCAN BIOSYNTHESIS”, “KEGG RIBOSOME”, “KEGG SPHINGOLIPID METABOLISM”, “KEGG FC GAMMA R MEDIATED PHAGOCYTOSIS” and “KEGG TGF BETA SIGNALING PATHWAY”

Among the 3 hub genes, TMEM45A was the only one that was up-regulated both in AF and SLE datasets. Thus, TMEM45A was chosen for further mechanistic studies in AF. The result of GSEA in AF showed that 16 pathways were enriched totally (Fig. 7A), with the top 3 pathways being “KEGG N GLYCAN BIOSYNTHESIS”, “KEGG RIBOSOME” and “KEGG SPHINGOLIPID METABOLISM” (Fig. 7B–D). Additionally, “KEGG FC GAMMA R MEDIATED PHAGOCYTOSIS” and “KEGG TGF BETA SIGNALING PATHWAY” were also enriched (Fig. 7E, F).

### The dynamic expression of TMEM45A in AF fibrosis and SLE patients

**Fig. 8:**
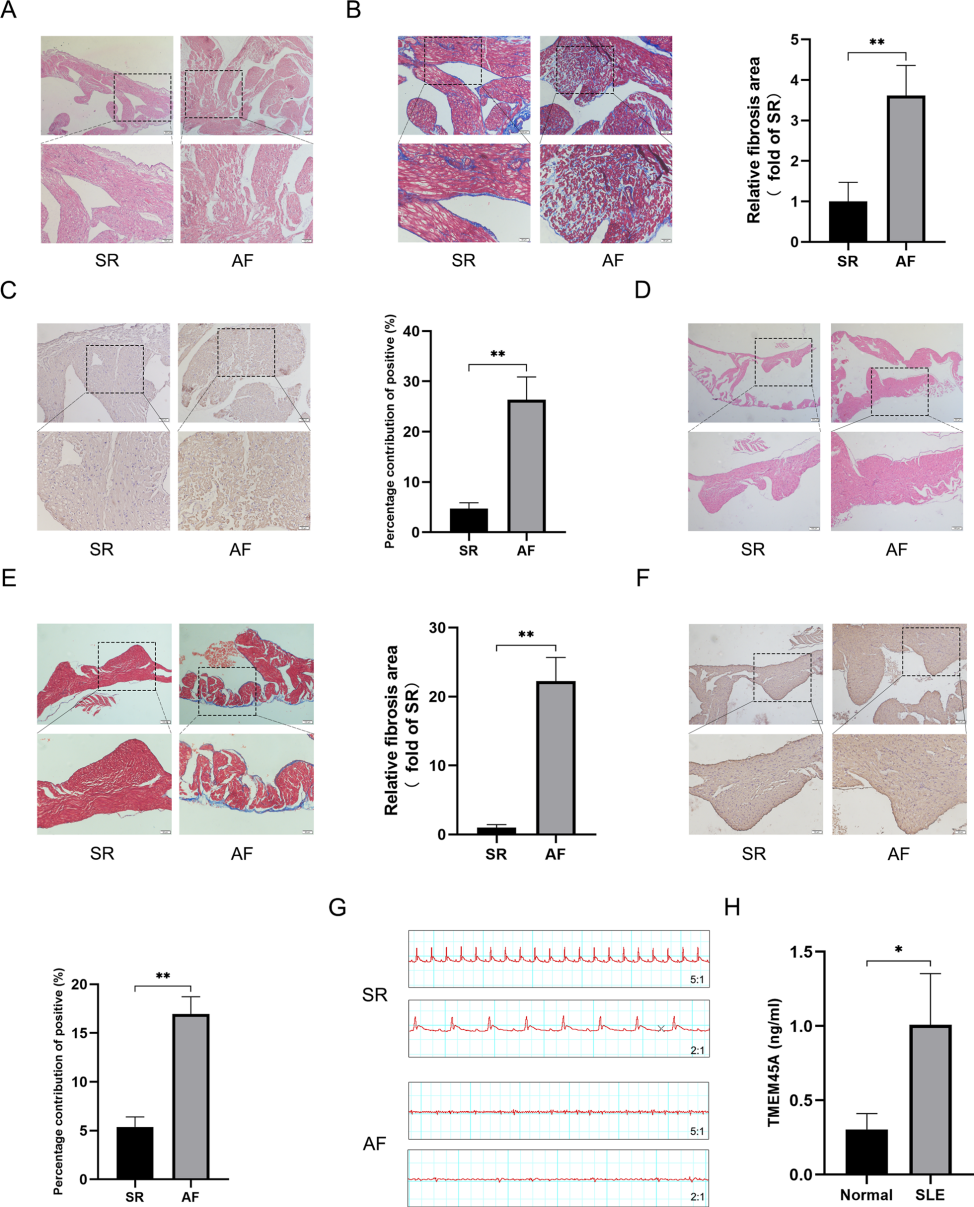
The validation of dynamic TMEM45A expression in AF and SLE. **A** The HE staining of the left atrium from normal and AF patients. **B** Representative Masson images and relative densitometric analysis of left atrium from normal (n = 10) and AF (n = 10) patients. **C** Representative immunohistochemistry images and relative densitometric analysis of TMEM45A in left atrium from normal (n = 10) and AF patients (n = 10). **D** The HE staining of the left atrium from normal and AF model constructed in rats. **E** Representative Masson images and relative densitometric analysis of left atrium from normal and AF model constructed in rats. **F** Representative immunohistochemistry images and relative densitometric analysis of TMEM45A in left atrium from normal and AF model constructed in rats. **G** Representative electrocardiogram from normal and AF model constructed in rats. **H** The aberrant expression of TMEM45A in SLE patients’ blood. **p < 0.01

Atrial samples from 10 normal and 10 AF patients were obtained, and HE staining results showed that atrial tissues in AF were disorganized arrangement, presenting hypertrophy and widened cell gaps in atrial myocytes (Fig. 8A). Masson staining revealed that the numbers of collagen fibers were significantly increased in AF atrium (Fig. 8B). Moreover, IHC results indicated significantly upregulation of TMEM45A expression in AF atrium (Fig. 8C). Furthermore, AF model in rats were constructed, with electrocardiogram results confirming the successful establishment of the model (Fig. 8G). HE staining results demonstrated that atrial myocytes in rat AF model presented significant hypertrophy (Fig. 8D). Additionally, significant fibrosis was also detected in the AF atrium of the rat model (Fig. 8E). Importantly, TMEM45A expression was up-regulated significantly in the rat AF atrium (Fig. 8F). Thus, the aberrant expression of TMEM45A was demonstrated in AF in vivo. Furthermore, the levels of TMEM45A in the blood of SLE patients were detected, and the expression of TMEM45A was significantly up-regulated in SLE patients (Fig. 8H).

### TMEM45A knockdown reduced AF inducibility

**Fig. 9:**
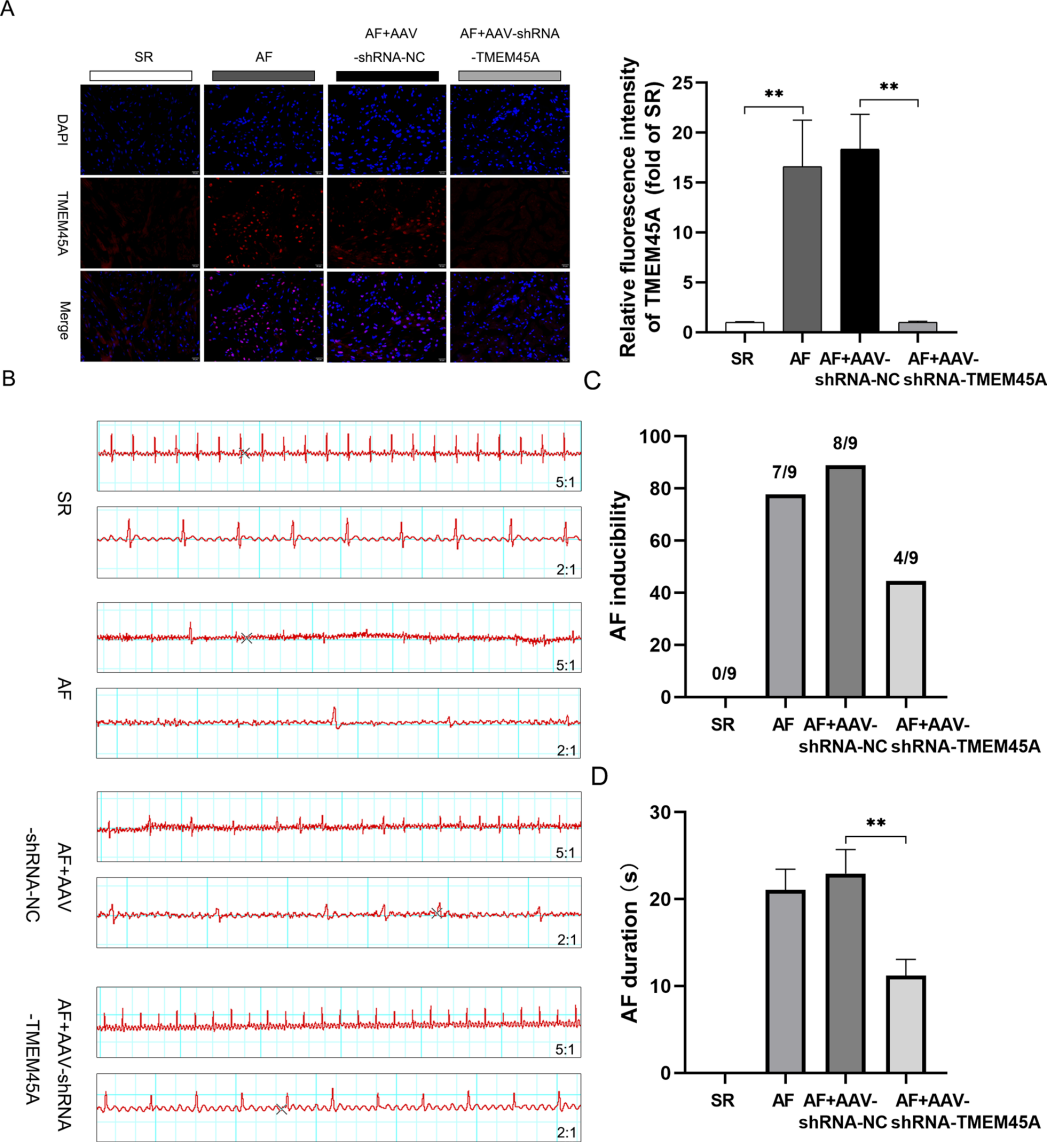
Knockdown of TMEM45A reduces AF inducibility. The (**A**) Representative immunofluorescence images and relative densitometric analysis of TMEM45A protein in rat atrial tissues. **B** Representative electrocardiogram from rats in different groups. **C**, **D** AF inducibility and AF durations in AF rats. *p < 0.05 and **p < 0.01

To verify the role of TMEM45A in AF, AAV-shRNA-TMEM45A was constructed and injected via jugular vein to induce the TMEM45A knockdown. IF analysis of TMEM45A in the the rat atrium confirmed the efficacy of AAV-shRNA-TMEM45A (Fig. 9A). After establishing the rat AF model, electrocardiograms for rats across different groups were recorded after inducing (Fig. 9B). Results indicated that AF inducibility and AF duration increased in the AF group and the AF + AAV-shRNA-NC group, but decreased significantly in the AF + AAV-shRNA-TMEM45A group **(**Fig. 9C, D). These results underscore the protective role of TMEM45A knockdown in AF.

### TMEM45A knockdown alleviated atrial fibrosis and collagen deposition in AF

**Fig. 10:**
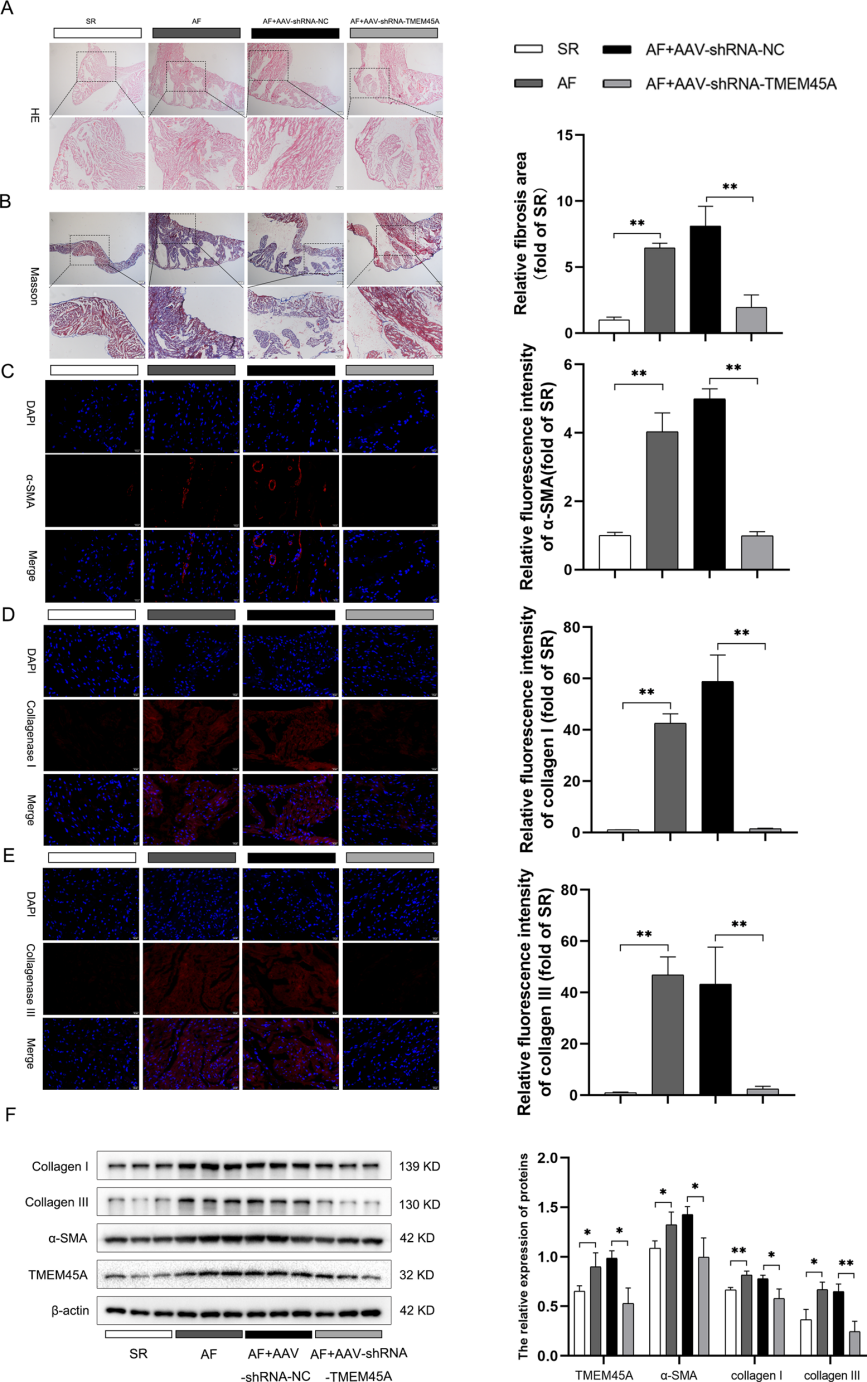
Knockdown of TMEM45A alleviated atrial fibrosis and collagen deposition in AF. **A** The HE staining of atrial tissues in rats. **B** Representative Masson images and relative densitometric analysis of atrial tissues in rats. **C** Representative immunofluorescence images and relative densitometric analysis of α-SMA protein in rat atrial tissues. **D** Representative immunofluorescence images and relative densitometric analysis of collagenase I protein in rat atrial tissues. **E** Representative immunofluorescence images and relative densitometric analysis of collagenase III protein in rat atrial tissues. **F** Representative western blot images and relative densitometric analysis of TMEM45A, collagen I, collagen III and α-SMA protein of atrial tissues in rats (n = 3). *p < 0.05 and **p < 0.01

To further verify the protective role of TMEM45A in AF, pathological alteration was examined in different groups. HE staining results revealed that atrial myocytes presented significant hypertrophy in the AF group and the AF + AAV-shRNA-NC group, while it was alleviated in AF + AAV-shRNA-TMEM45A group (Fig. 10A). Furthermore, masson staining results indicated deteriorated fibrosis in the AF group and the AF + AAV-shRNA-NC group was mitigated in the AF + AAV-shRNA-TMEM45A group (Fig. 10B). Additionally, atrial fibrosis was characterized by excessive ECM (extracellular matrix) deposition. Thus, the expression of fibrosis markers, including collagenase I, collagenase III and α-SMA, was assessed in rat atrial tissues through IF. Results indicated that the expression of collagenase I, collagenase III and α-SMA were significantly increased in the AF group and the AF + AAV-shRNA-NC group, while they decreased in the AF + AAV-shRNA-TMEM45A group (Fig. 10C–E). Additionally, western blots results confirmed that the increased expression of collagenase I, collagenase III and α-SMA were rescued following TMEM45A knockdown (Fig. 10F). Taken together, these findings demonstrated that TMEM45A knockdown alleviated atrial fibrosis and ECM deposition.

### TMEM45A knockdown inhibited NRCFs activation through TGF-β/smad2/3 pathway

**Fig. 11:**
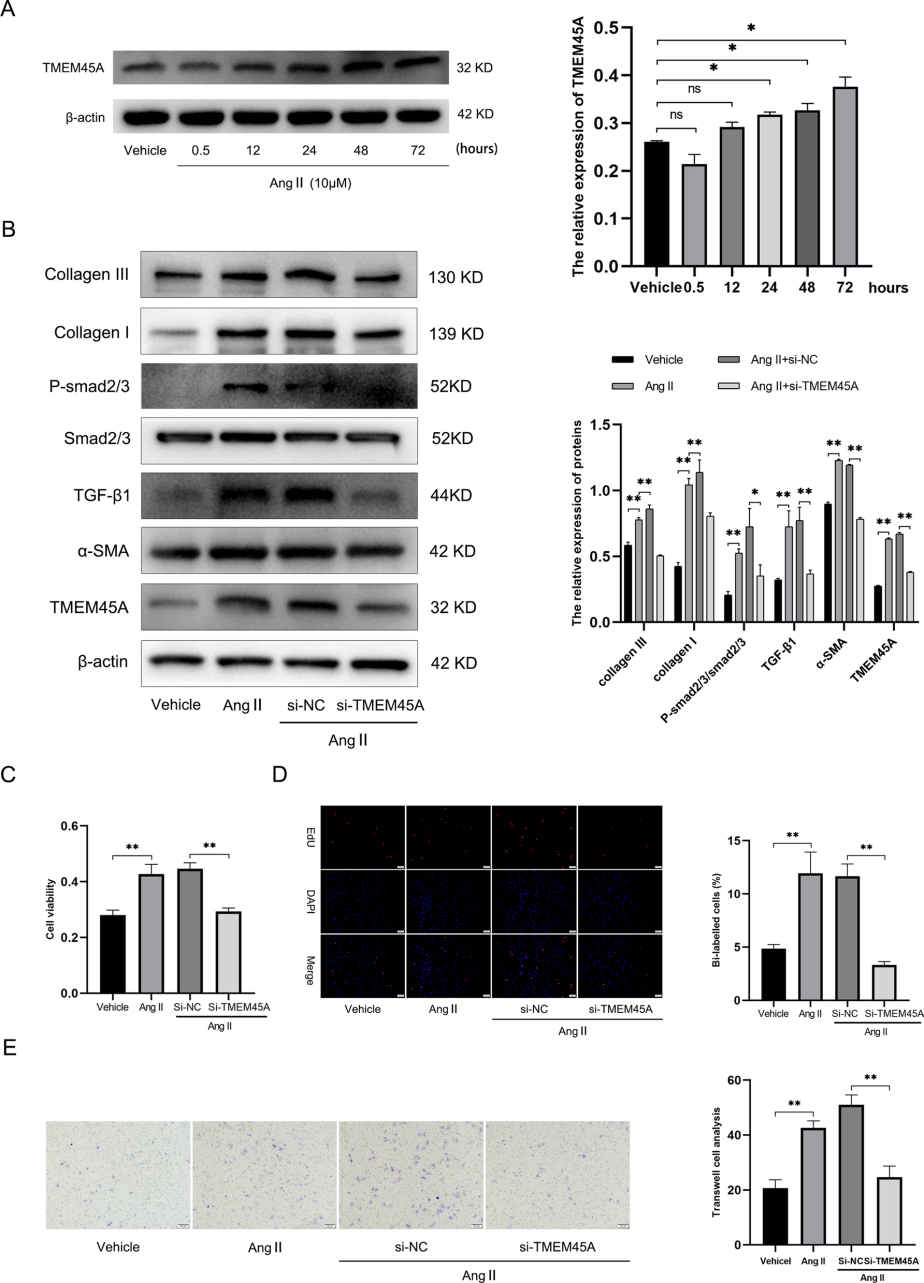
Knockdown of TMEM45A inhibited NRCFs activation through TGF-β/smad2/3 pathway. **A** Representative western blot images and relative densitometric analysis of TMEM45A protein in NRCFs after exposure to Ang II for different durations. **B** Representative western blot images and relative densitometric analysis of TMEM45A, collagen I, collagen III, α-SMA, TGF-β and p-smad2/3 protein in NRCFs transfected with si-TMEM45A under Ang II stimulation. β-Actin was used as a loading control. **C** Cell proliferation ability was measured by CCK8 assay. **D** EdU incorporation in VSMCs, the EdU-positive signal (purple) was merged with nuclei (blue) stained with Hoechst 33342. EdU-positive cells were quantified by Image-Pro Plus. **E** VSMC migration ability was measured by Transwell assays (n = 3), and migrated cells were quantified by Image-Pro Plus. *p < 0.05 and **p < 0.01

Furthermore, angiotensin II (Ang II) was used to establish the NRCFs fibrosis model in vitro. The expression of TMEM45A was upregulated obviously after 72 h of stimulation (Fig. 11A). Subsequently, the siRNA targeting TMEM45A was constructed and transfected into NRCFs. After the inhibiting of TMEM45A expression in Ang II-induced NRCFs, fibrosis biomarkers including collagen I, collagen III and α-SMA were obviously down-regulated (Fig. 11B). Furthermore, the biological function of NRCFs was determined. CCK-8 assay on cell viability manifested that TMEM45A knockdown reduced Ang II-induced NECFs’ proliferation (Fig. 11C). Result from the EdU assay corroborated the results of CCK-8 assay, showing that the number of EdU-positive cells was significantly suppressed in the si-TMEM45A + Ang II group compared with the si-NC + Ang II group (Fig. 11D). Differences in migration ability among the groups were observed through Transwell assay (Fig. 11E). Moreover, western blot result indicated that Ang II-induced expression of collagen I, collagen III and α-SMA decreased after TMEM45A knockdown in NRCFs. In addition, the elevated expression of TGF-β and p-smad2/3 was also decreased following TMEM45A knockdown. Thus, it was indicated that TMEM45A knockdown inhibited NRCFs activation through TGF-β/smad2/3 pathway.

## Discussion

Benefiting from the widespread application of methods of microarray and bioinformatic analysis, the potential landscape and mechanism underlying miscellaneous diseases can be investigated. AF is the most common cardiac arrhythmia in the general population, with patients with SLE facing a higher risk (Yafasova et al. 2021). However, the underlying gene variations and patterns in AF patients combined with SLE remain unclear. Thus, utilizing extensive databases, prospective therapeutic targets were explored to illuminate the mechanisms of AF combined with SLE. In the present study, 3 upregulated targets, including TMEM45A, ITGB2 and NFKBIA, were identified through machine learning approaches.

Among the hub genes, NFKBIA presented a close association with AF combined with SLE. Previous genome-wide association studies have indicated that NFKBIA was a potential fundamental gene implicated in SLE development (Gorji et al. 2019; Li et al. 2013). NFKBIA and its protein product IκBα are part of the regulatory loop, wherein NFKBIA transcription is activated by NF-κB, while IκBα acted as the primary inhibitor of NF-κB activation (Paciolla et al. 2011). Polymorphisms in the IκBα gene were found to affect the development and clinical manifestation of SLE (Lin et al. 2008). Though there was no direct evidence reflecting the association between NFKBIA and AF development, the activation of NF-κB signaling pathway was known to contribute to atrial remolding and AF vulnerability (Hsu et al. 2023). In the present study, NFKBIA was found to be up-regulated both in SLE and AF, suggesting a potential diagnostic value for AF combined with SLE.

ITGB2 (Integrin Subunit Beta 2) encodes an integrin beta chain (CD18) and combines with multiple different alpha chains to form different Beta-2-integrins, playing a pivotal role in leukocyte adhesion and immune inflammation (Tan 2012). Though ITGB2 expression presented no significant difference in our analysis of SLE dataset DEGs, comprehensive bioinformatics analyses have indicated that ITGB2 was a potential biomarker for SLE and may affect the process of Neutrophil extracellular traps in SLE (Li et al. 2023). Similarly, the result of bioinformatic research suggested that ITGB2 might be a potential therapeutic target for anti-inflammatory treatments in AF (Ying et al. 2023), though the underlying mechanism remained unknown. It has been demonstrated that activation of ITGB2 transcription can regulate macrophage tracking and contribute to myocardial fibrosis (Liu et al. 2022). Thus, aberrant ITGB2 expression suggested crosstalk between inflammatory responses and cardiac fibrosis in AF combined with SLE.

Importantly, elevated expression of TMEM45A was both predicted and validated in the atrium of AF samples. The present study identified TMEM45A as a risk gene in SLE-related AF, and demonstrated its aberrant expression in the atrium of AF patients. Furthermore, in the rat model of AF, we demonstrated TMEM45A knockdown alleviated AF inducibility and atrial fibrosis. Previous study has demonstrated that TMEM45A was involved in the pathology of renal fibrosis (Lan et al. 2020). Our findings confirmed that TMEM45A promoted cardiac fibroblast fibrosis, potentially elucidating its role in AF development. Based on the result of GSEA of TMEM45A in the AF dataset, TGF-β signaling pathway was enriched. Our results confirmed that TMEM45A may affect cardiac fibrosis via TGF-β signaling pathway. As a transmembrane protein, TMEM45A almost achieve biological function by binding to ligands. Identifying TMEM45A’s ligand and elucidating the complete signaling pathway affecting cardiac fibrosis may pave the way for future research.

Available evidence has indicated the relationship between the development of AF and systemic inflammation in autoimmune diseases including SLE (Lin et al. 2022). Previous study has reported that local cardiac inflammatory conditions increased the incidence of AF (Hu et al. 2015). In SLE, myocardial involvement is common, with an average prevalence of myocarditis in 50%-80% of SLE patients (Apte et al. 2008). In the present study, through immune cell infiltration analysis with ssGSEA, the common upregulated immune cells in AF and SLE included activated CD4 T cell, activated dendritic cell, central memory CD8 T cell, immature dendritic cell, macrophage, mast cell, MDSC and neutrophile. Most major immune cell subpopulations were identified in the diseased heart including AF (Martini et al. 2019). In addition, accumulative evidence has unveiled the dynamic immune cell profiles in the circulatory system of SLE (Tsokos et al. 2016). The hub genes NFKBIA and ITGB2 were both significantly related to immune cell function in AF and SLE, implying these hub genes could contribute to the occurrence of AF. Thus, SLE may create an immune microenvironment that can cause organ damage, including local myocarditis, leading to AF onset. As a result, elucidating the molecular mechanisms underlying SLE’s immunological profiles could illuminate therapeutic intervention for AF.

It has been surmised that the inflammatory and immune processes contributed to AF development, subsequently generating an inflammatory response that exacerbated cardiac remolding and perpetuate the arrhythmia (Hu et al. 2015). It was acknowledged that active autoimmune system in SLE was characterized by various pro-inflammatory cytokines in peripheral blood. GO enrichment in our study revealed that signaling pathways including “cytokine-mediated signal pathway”, “receptor ligand activity” and “secretory granule membrane” were enriched, further indicating that autoimmune response in SLE may be the potential underlying mechanisms of AF initiation in SLE. In addition, KEGG enrichment analysis identified pathways such as the “IL-17 signaling pathway”, “TNF signaling pathway” and “Rheumatoid arthritis”. It was speculated that auto-inflammatory response may be linked to the development of arrhythmias, with AF being one of the most common (Lin et al. 2022). Therefore, inflammatory pathways may account for the pathological mechanism of SLE-associated AF.

Despite the ability of current therapies for the management of AF to improve clinical outcomes, pharmacological rhythm-control therapies are limited due to potential proarrhythmic side effects and individual mechanisms of AF. Moreover, none of the currently used drugs were specific to target AF based on the underlying arrhythmia mechanisms (Saljic et al. 2022). Thus, it is imperative to identify the potential drugs based on the AF-related targets and signaling pathways. In the present study, cMAP analysis of core genes associated with AF and SLE identified small-molecular targeted compounds. Of the top 10 compounds, besides ouabain, digoxin, digitoxin and proscillaridin which were already applied in clinic for AF management, other potential drugs including ryuvidine, KI-8751, iodoacetic-acid, YM-155, BX-795 and CS-110266 were obtained. Among these drugs, KI-8751 was an inhibitor of VEGFR, which has been demonstrated to be involved in the pathophysiology of AF (Chang et al. 2021). Moreover, YM-155 has been reported to have the ability to ameliorate cellular fibrosis (Wu et al. 2023), which may affect cardiac fibrosis. Thus, it was speculated that KI-8751 and YM-155 could prevent the initiation and progression of AF.

The present study identified crosstalk genes in AF and SLE, thus providing new insights into the co-morbidity mechanisms between AF and SLE. Crucially, TMEM45A-induced cardiac fibrosis was demonstrated to contribute to AF development in vivo and in vitro. However, limited by transcriptome sequencing data, the correlation between TMEM45A and immune cells in AF was not clear. Though the role of TMEM45A in NRCFs fibrosis was demonstrated, the deeper mechanisms involving TMEM45A in AF development was not unraveled. Additionally, it should be noted that the AF model in vivo and in vitro was not related to SLE in the study. Therefore, establishing SLE-induced AF model may lend more persuasiveness and credibility to the study’s findings.

## Conclusion

In summary, three crosstalk genes including TMEM45A, ITGB2 and NFKBIA were identified in AF and SLE. Furthermore, TMEM45A-induced cardiac fibrosis was demonstrated to contribute to AF development both in vivo and in vitro. This study provided new insights into the co-pathogenesis of AF and SLE and identified new targets for AF prevention and treatment.

## Supplementary Information

Additional file 1: Figure 1. The strategy of bioinformatics analysis.

Additional file 2: Figure 2. The integration of AF datasets. (A) The PCA of original AF datasets. (B) The PCA of the integrated AF datasets after batch-effect correction.

Additional file 3: Figure 3. The immune cell infiltration in AF. (A) The heatmap of 28 immune cells expression in AF. (B) The comparison of 28 immune cells infiltration in samples of control and AF. (C, D, E) The correlation between immune cells abundance and hub genes including TMEM45A, ITGB2 and NFKBIA in AF.

Additional file 4: Figure 4. The immune cell infiltration in SLE. (A) The heatmap of 28 immune cells expression in SLE. (B) The comparison of 28 immune cells infiltration in samples of control and SLE. (C, D, E) The correlation between immune cells abundance and hub genes TMEM45A, ITGB2 and NFKBIA in SLE.

Additional file 5.

## Data Availability

Data are available upon reasonable request. The analysis code has been deposited on GitHub (https://github.com/xuhongjie1224/demo) and related explanation can be also obtained on GitHub (https://github.com/xuhongjie1224/demo/wiki).
