# Supplementary figures and images for: Integrated bioinformatics and validation reveal TMEM45A in systemic lupus erythematosus regulating atrial fibrosis in atrial fibrillation
Source: Mol Med. 2025 Mar 18;31:104. doi: 10.1186/s10020-025-01162-0 (PMC11917082; doi:10.1186/s10020-025-01162-0)

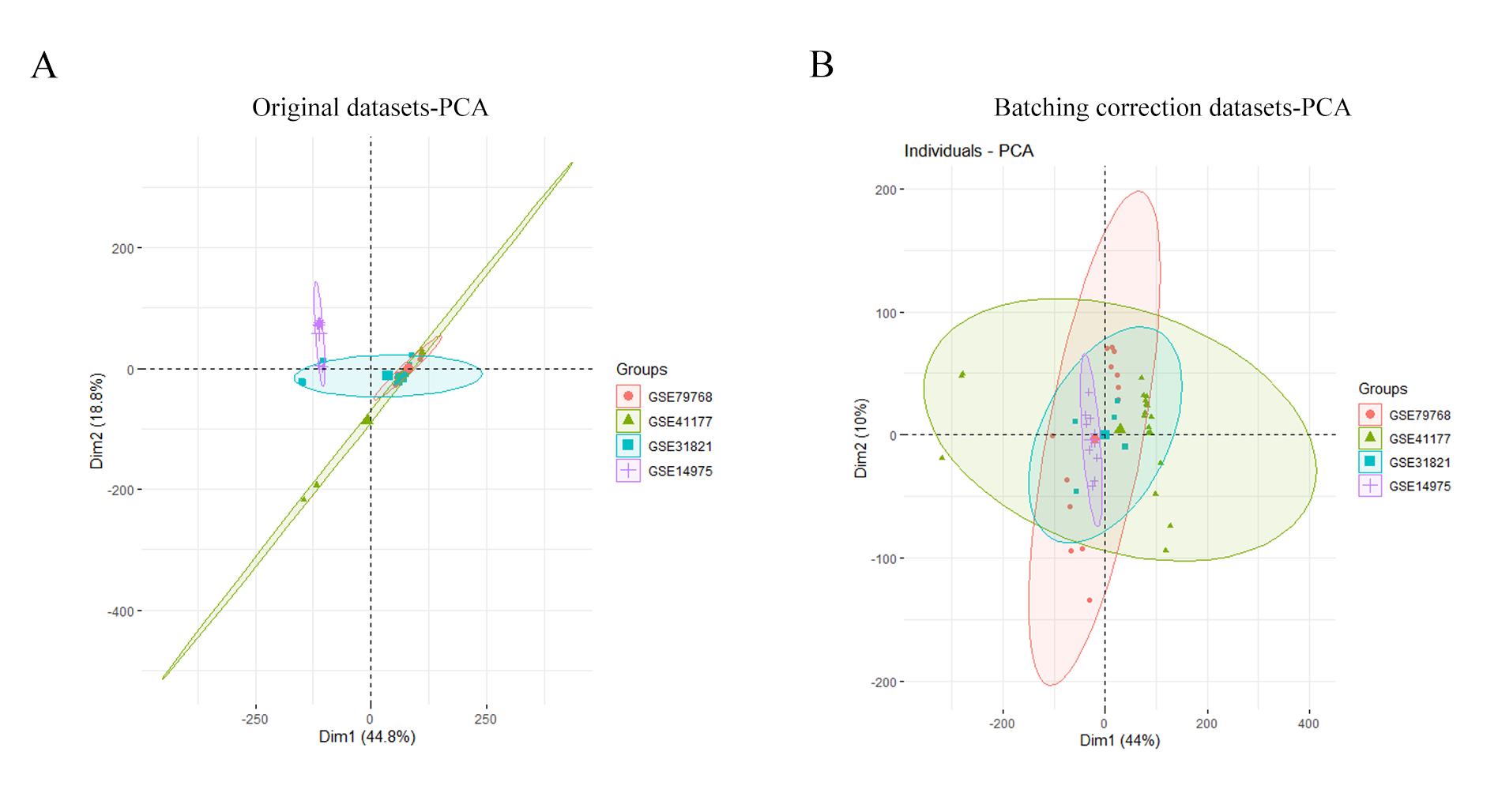

Supplement: Supplementary file 2 — Additional file 2: Figure 2. The integration of AF datasets. (A) The PCA of original AF datasets. (B) The PCA of the integrated AF datasets after batch-effect correction. [file 10020_2025_1162_MOESM2_ESM.tif]

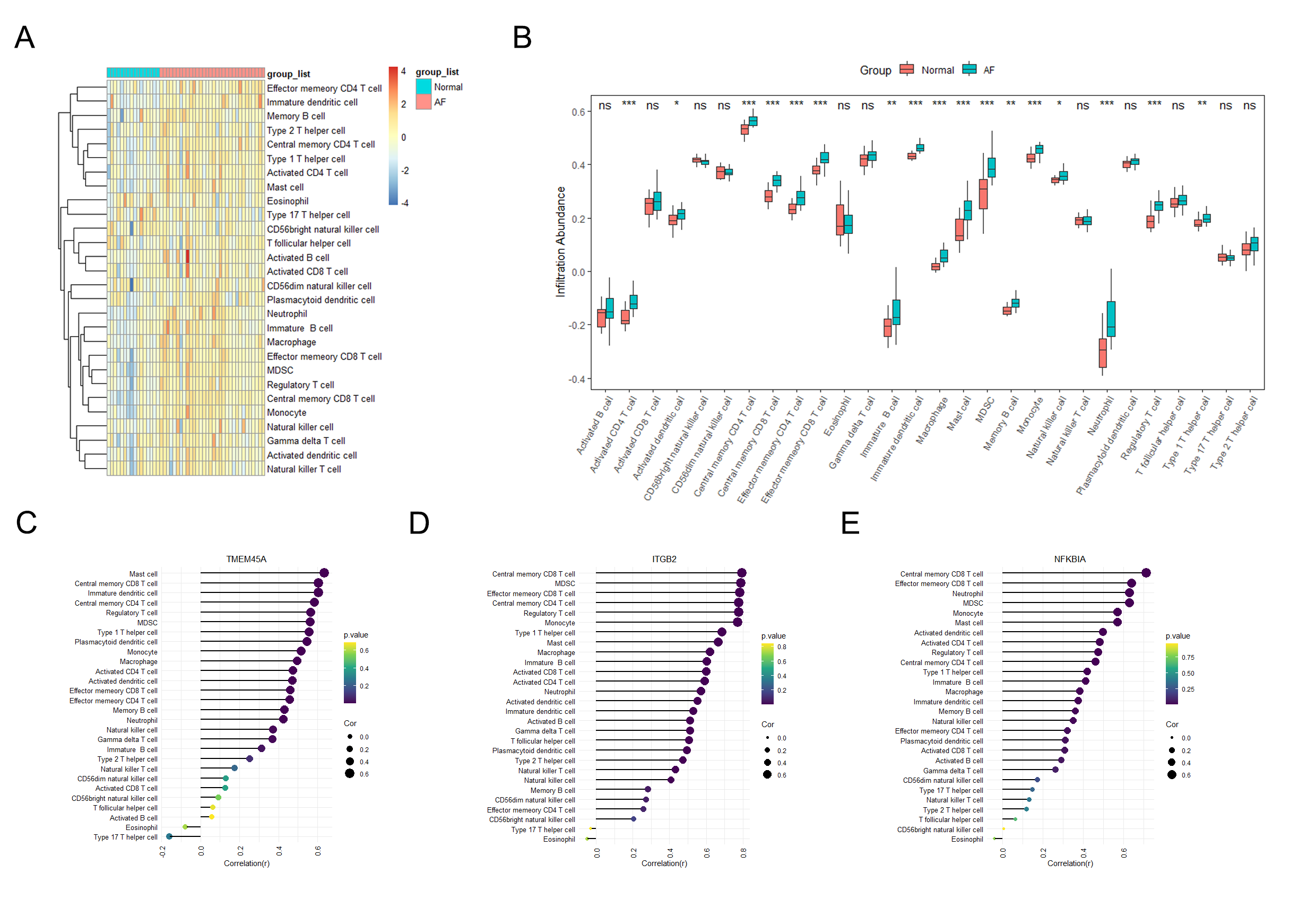

Supplement: Supplementary file 3 — Additional file 3: Figure 3. The immune cell infiltration in AF. (A) The heatmap of 28 immune cells expression in AF. (B) The comparison of 28 immune cells infiltration in samples of control and AF. (C, D, E) The correlation between immune cells abundance and hub genes including TMEM45A, ITGB2 and NFKBIA in AF. [file 10020_2025_1162_MOESM3_ESM.tif]

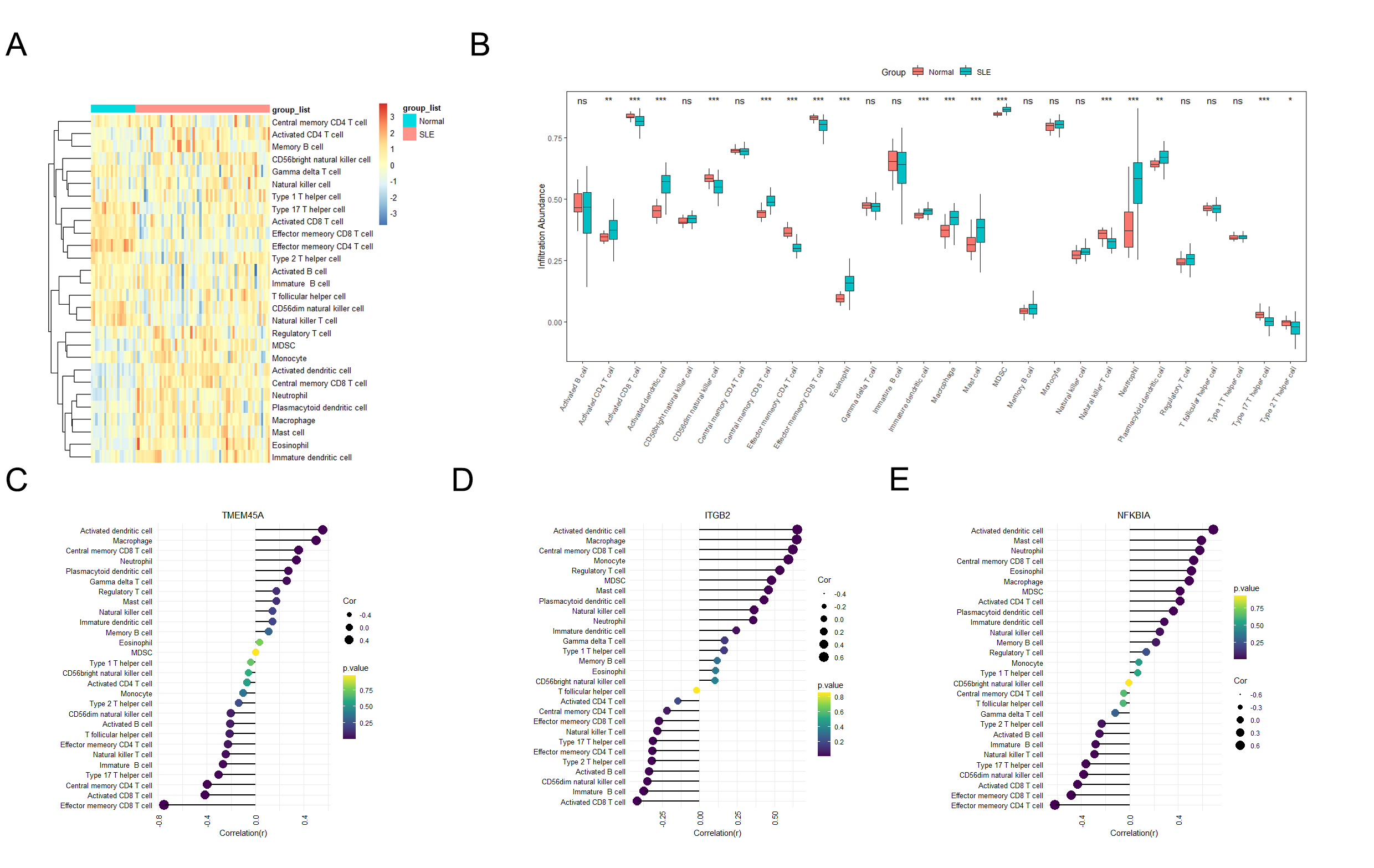

Supplement: Supplementary file 4 — Additional file 4: Figure 4. The immune cell infiltration in SLE. (A) The heatmap of 28 immune cells expression in SLE. (B) The comparison of 28 immune cells infiltration in samples of control and SLE. (C, D, E) The correlation between immune cells abundance and hub genes TMEM45A, ITGB2 and NFKBIA in SLE. [file 10020_2025_1162_MOESM4_ESM.tif]
