# Supplementary material for: Integrated bioinformatics and validation reveal TMEM45A in systemic lupus erythematosus regulating atrial fibrosis in atrial fibrillation
Source: Mol Med. 2025 Mar 18;31:104. doi: 10.1186/s10020-025-01162-0 (PMC11917082; doi:10.1186/s10020-025-01162-0)
Supplement: Supplementary file 5 — Additional file 5. [file 10020_2025_1162_MOESM5_ESM.docx]

Supplementary table 1 Clinical characteristics of patients

| Variables | SR(n=10) | AF(n=10) | P Value |
| --- | --- | --- | --- |
| Age, years | 57.5±3.5 | 63.3±2.8 | 0.210 |
| Female | 7(70.0) | 5(50.0) | 0.650 |
| BMI | 22.5±0.9 | 22.3±1.2 | 0.913 |
| Hypertension | 1(10.0) | 2(20.0) | 1.000 |
| Stroke | 0(0.0) | 1(10.0) | 1.000 |
| Diabetes | 3(30.0) | 1(10.0) | 0.582 |

^a^SR: sinus rhythm. AF: atrial fibrillation.

Supplementary table 2 The expression pattern of 26 DEGs in AF and SLE

|  | AF | | SLE | |
| --- | --- | --- | --- | --- |
| Symbol | logFC | P.Value | logFC | P.Value |
| BCL2A1 | >1.00 | 9.59E-05 | 1.32 | 7.92E-10 |
| C1QB | 1.12 | 4.90E-06 | 1.60 | 1.51E-13 |
| C1QC | 1.17 | 9.75E-09 | 1.56 | 7.41E-14 |
| CCL4 | 1.01 | 1.83E-07 | 1.20 | 2.41E-05 |
| CMTM2 | 1.36 | 6.40E-10 | 1.62 | 1.02E-09 |
| COG3 | 1.01 | 3.03E-08 | <-1.00 | 1.42E-08 |
| CXCR2 | 1.36 | 7.11E-06 | 1.91 | 8.24E-11 |
| CYBA | 1.05 | 6.65E-08 | 1.22 | 1.34E-32 |
| DHRS9 | 1.22 | 6.62E-05 | 1.16 | 2.78E-17 |
| DUSP1 | 1.32 | 1.12E-08 | 1.70 | 6.55E-22 |
| EGR1 | 1.17 | 0.000119 | 1.55 | 4.03E-08 |
| FAR1 | 1.03 | 7.30E-05 | -1.20 | 1.13E-08 |
| FCGR1BP | 1.06 | 9.79E-07 | 1.52 | 1.15E-14 |
| FCGR3B | 2.14 | 2.38E-13 | 2.60 | 5.16E-23 |
| FOS | 1.02 | 0.001391 | 2.21 | 2.39E-17 |
| IGLV3-25 | 1.07 | 3.09E-05 | 1.36 | 4.65E-06 |
| IL1R1 | >1.00 | 5.51E-08 | 1.52 | 1.15E-12 |
| MARF1 | 1.11 | 1.97E-09 | -1.20 | 8.95E-11 |
| PROK2 | 1.30 | 2.83E-07 | 1.76 | 6.14E-09 |
| PTGS2 | 1.24 | 1.71E-05 | 2.10 | 3.22E-07 |
| S100A12 | 1.57 | 1.10E-05 | 1.14 | 3.28E-11 |
| SORT1 | 1.19 | 2.23E-08 | -1.30 | 1.11E-15 |
| STAT1 | 1.34 | 6.88E-09 | 1.03 | 9.93E-11 |
| STEAP4 | >1.00 | 2.37E-05 | 1.16 | 2.20E-06 |
| TGFBR1 | 1.07 | 3.64E-06 | -1.10 | 7.47E-21 |
| TMEM45A | 1.09 | 5.51E-10 | 1.14 | 0.001626 |

^a^DEGs: differentially expressed genes. AF: atrial fibrillation, SLE: systemic lupus erythematosus.

Supplementary table 3 The expression pattern of 3 hub genes in AF datasets

|  | GSE14975 | | GSE31821 | | GSE79768 | | GSE41177 | |
| --- | --- | --- | --- | --- | --- | --- | --- | --- |
| Symbol | logFC | P.Value | logFC | P.Value | logFC | P.Value | logFC | P.Value |
| TMEM45A | 1.21 | 0.00015263 | 0.54^*^ | 0.313273 | 0.61 | 0.04946 | 1.88 | 8.30553E-08 |
| ITGB2 | 1.05 | 9.4528E-08 | 1.14 | 0.025933 | 1.02 | 0.00035 | 2.80 | 3.62751E-08 |
| NFKBIA | 0.71^*^ | 0.01892022 | 1.54 | 0.002238 | 0.43^*^ | 0.013193 | 2.67 | 1.4947E-06 |

^a^AF: atrial fibrillation. ^*^means that the value was less that one.

Supplementary table 4 The expression pattern of 3 hub genes in SLE dataset

|  | GSE50772 | |
| --- | --- | --- |
| Symbol | logFC | P.Value |
| TMEM45A | 1.14 | 0.001626 |
| ITGB2 | 0.45^*^ | 1.13E-05 |
| NFKBIA | 1.86 | 2.48E-19 |

^a^SLE: systemic lupus erythematosus. ^*^means that the value was less that one.

Supplementary table 5 The expression pattern of 3 hub genes in merged AF dataset

| Symbol | logFC | P.Value |
| --- | --- | --- |
| TMEM45A | 1.091 | 5.5077E-10 |
| ITGB2 | 1.374 | 1.0184E-11 |
| NFKBIA | 0.61^*^ | 3.4975E-12 |

^a^AF: atrial fibrillation. ^*^means that the value was less that one.
